# The Secretory Pathway Calcium ATPase PMR-1/SPCA1 Has Essential Roles in Cell Migration during *Caenorhabditis elegans* Embryonic Development

**DOI:** 10.1371/journal.pgen.1003506

**Published:** 2013-05-16

**Authors:** Vida Praitis, Jeffrey Simske, Sarah Kniss, Rebecca Mandt, Leah Imlay, Charlotte Feddersen, Michael B. Miller, Juliet Mushi, Walter Liszewski, Rachel Weinstein, Adityarup Chakravorty, Dae-Gon Ha, Angela Schacht Farrell, Alexander Sullivan-Wilson, Tyson Stock

**Affiliations:** 1 Biology Department, Grinnell College, Grinnell, Iowa, United States of America; 2 Rammelkamp Center for Education and Research, Case Western Reserve University, Cleveland, Ohio, United States of America; 3 Molecular Genetics and Cell Biology, University of Chicago, Chicago, Illinois, United States of America; University of California San Diego, United States of America

## Abstract

Maintaining levels of calcium in the cytosol is important for many cellular events, including cell migration, where localized regions of high calcium are required to regulate cytoskeletal dynamics, contractility, and adhesion. Studies show inositol-trisphosphate receptors (IP3R) and ryanodine receptors (RyR), which release calcium into the cytosol, are important regulators of cell migration. Similarly, proteins that return calcium to secretory stores are likely to be important for cell migration. The secretory protein calcium ATPase (SPCA) is a Golgi-localized protein that transports calcium from the cytosol into secretory stores. SPCA has established roles in protein processing, metal homeostasis, and inositol-trisphosphate signaling. Defects in the human SPCA1/ATP2C1 gene cause Hailey-Hailey disease (MIM# 169600), a genodermatosis characterized by cutaneous blisters and fissures as well as keratinocyte cell adhesion defects. We have determined that PMR-1, the *Caenorhabditis elegans* ortholog of SPCA1, plays an essential role in embryogenesis. Pmr-1 strains isolated from genetic screens show terminal phenotypes, such as ventral and anterior enclosure failures, body morphogenesis defects, and an unattached pharynx, which are caused by earlier defects during gastrulation. In Pmr-1 embryos, migration rates are significantly reduced for cells moving along the embryo surface, such as ventral neuroblasts, C-derived, and anterior-most blastomeres. Gene interaction experiments show changing the activity of *itr-1*/IP3R and *unc-68*/RyR modulates levels of embryonic lethality in Pmr-1 strains, indicating *pmr-1* acts with these calcium channels to regulate cell migration. This analysis reveals novel genes involved in *C. elegans* cell migration, as well as a new role in cell migration for the highly conserved SPCA gene family.

## Author Summary

During growth or regeneration after damage, skin cells migrate from basal to superficial layers, forming tight attachments that protect an individual from environmental assaults. Proteins that remove calcium from the cell cytosol into secretory stores, where it is available for future release, play a key role in skin cell integrity. Defects in these secretory pathway calcium ATPase (SPCA) channels in humans cause Hailey-Hailey disease, a chronic disorder marked by skin lesions in areas of high-stress. Our study of the SPCA gene *pmr-1* in *Caenorhabditis elegans* indicates the gene is essential for viability. Embryos with defective PMR-1 die with cell attachment defects superficially similar to those of Hailey-Hailey disease patients. To better understand this phenotype, we tracked the position of individual cells during development of *pmr-1* mutant embryos. This analysis revealed that the cell attachment defects are caused by primary failures in cell migration. We also identified other calcium channel proteins involved in this process, indicating proper regulation of calcium is crucial for cell migration in *C. elegans*. If SPCA proteins act similarly in humans, this research will lead to better understanding of the molecules important for skin cell regeneration, as well as help to explain the defects observed in Hailey-Hailey disease patients.

## Introduction

Calcium is one of the most versatile and important small molecules in the cell: it is involved in cell processes starting at the beginning of new cell life during fertilization and ending with a role in cell death during apoptosis [1]. One developmental role for calcium is in cell migration. Studies have determined that migrating cells establish a relatively stable calcium gradient, with higher levels in the rear of the cell [2]. The increased calcium levels at the rear of the cell are likely important for regulating cell detachment and contraction [3], [4]. More recently, studies identified transient flickers of high calcium enriched at the leading edge of migrating cells and preceding directional changes. These calcium flickers may provide localized cues that help direct migration [4]–[6].

Calcium-sensitive molecules important for cell migration can be divided into two broad categories: those that sense and respond to differences in calcium levels and those that control levels of calcium by transporting it across membranes [1]. In this second class, channels that release calcium into the cytosol, such as the plasma-membrane-associated transient receptor potential (TRP) channels, partnering with the secretory pathway inositol-trisphosphate channels (IP3R) and ryanodine receptors (RyR), have been shown to play a key role in producing calcium flickers and in cell migration [1], [4], [7], [8]. The localized movement of calcium out of the cytosol must also be important for producing calcium flickers and gradients. Calcium uptake from the cytosol requires Ca^2+^ ATPase transporters found at three distinct subcellular locations: the plasma membrane (PMCA), the Sarco(endo)plasmic reticulum (SERCA) and the secretory pathway/Golgi (SPCA) [1], [9], [10]. SERCA, which localizes to both the endoplasmic reticulum (ER) and Golgi, helps to control cytosolic calcium levels in a variety of cells and is integral for proper IP3 signaling [1]. The Golgi-localized SPCA, which has not been as extensively characterized, transports both Ca^2+^ and Mn^2+^, playing an important role in homeostasis for both molecules. SPCA is also critical for protein processing in the Golgi, Ca^2+^ release in response to IP3 signaling, and stress tolerance [1], [9], [11]. While at least one of these Ca^2+^ ATPase proteins is likely to be involved in maintaining subcellular calcium gradients important for cell migration, this link has yet to be firmly established.

Genetic studies show the highly conserved SPCA1 protein has essential roles in embryonic development and disease. Loss of a single copy of the SPCA1 gene in humans causes Hailey-Hailey disease (MIM# 169600), a skin disorder characterized by recurrent lesions or blisters of the skin in areas subject to high stress [12]–[16]. SPCA1 is likely essential in humans, as more severe phenotypes are found in patients who suffer clonal loss of both copies of the gene [17], [18]. Mice embryos homozygous for null mutations in SPCA1 die with defects in neural tube closure, while heterozygotes show susceptibility to squamous cell tumors, a phenotype observed occasionally in humans with Hailey-Hailey [19]–[22]. The primary cellular defect in Hailey-Hailey disease patients is keratinocyte acantholysis or loss of cell adhesion, marked by aggregation of keratin intermediate filaments that have retracted from desmosomes [23], [24]. In mice, cell adhesion appears normal, but cells show signs of Golgi dysfunction, which likely induces the high levels of apoptosis observed in the neural tube and mesenchyme [19]. Based on this genetic analysis, it is not evident that SPCA, which has a conserved role at the molecular level, has a similarly conserved role in development, although each phenotype may be the result of secretory pathway stress [25].

*Caenorhabditis elegans* has served as a good model system for studying calcium signaling because it has the full complement of membrane-associated calcium channels. Research has revealed a role for the calcium channel ITR-1/IP3R in ovulation, gastrulation, pharyngeal pumping, defecation and other developmental processes [26]. It is expressed in the embryo and is the only channel shown to play a direct role in cell migration, as *itr-1* mutants fail to complete ventral closure during *C. elegans* development [26]–[28]. The UNC-68/RyR localizes to both ER and Golgi and plays a key role in muscle function [7], [29]–[32]. The single SPCA1 gene, *pmr-1*, encodes a protein that, like its vertebrate counterpart, co-localizes with Golgi markers, when expressed in COS cells or in *C. elegans* [33]–[35]. Based on RNA *in situ* hybridization and microarray analysis, *pmr-1* is expressed in the adult germ line and at moderate levels throughout embryogenesis in most blastomeres [36]–[38]. *pmr-1* transcriptional and translational fusion constructs show expression starting from the 3-fold embryo and continuing until the adult stage in the nervous system, intestine, intestinal valve, hypodermis, spermatheca, and gonad [35], [39]. The absence of germ line and early embryo expression for these fusion constructs may indicate they are silenced [40] or contain incomplete regulatory sequences. PMR-1 protein also shows functional conservation, with important roles in Ca^2+^ spiking in response to IP3 signaling, stress response, thermotolerance, pathogen resistance, and metal homeostasis [26], [33]–[35], [41], [42].

Using alleles of *pmr-1* obtained in forward genetic and deletion screens, we have identified a new, essential role for *pmr-1* in embryonic development in *C. elegans*. Strains homozygous for mutations in *pmr-1* show temperature-sensitive embryonic lethality caused by defects in ventral enclosure, apical enclosure, and morphogenesis. By identifying the temperature-sensitive period we have learned that *pmr-1* plays an important role during the period preceding ventral enclosure. While *pmr-1* mutants show normal cell fate specification, lineage, and cell ingressions during gastrulation, we see defects in the migration of cells along the surface of embryo following ingression in the C-lineage, ventral neuroblasts, and in anterior lineages, a cell migration process that appears similar to migrations during neurulation in vertebrates. Phenotypes in *pmr-1* mutants can be suppressed or enhanced by changes in the activity of the RyR *unc-68* and IP3R *itr-1* genes, indicating that these cell migrations are sensitive to calcium levels. This study identifies new molecules important for embryonic cell migration in *C. elegans* and reveals a new role in cell migration for the calcium channel PMR-1/SPCA protein family.

## Results

### Isolation and genetic analysis of *pmr-1* alleles

**Figure 1:**
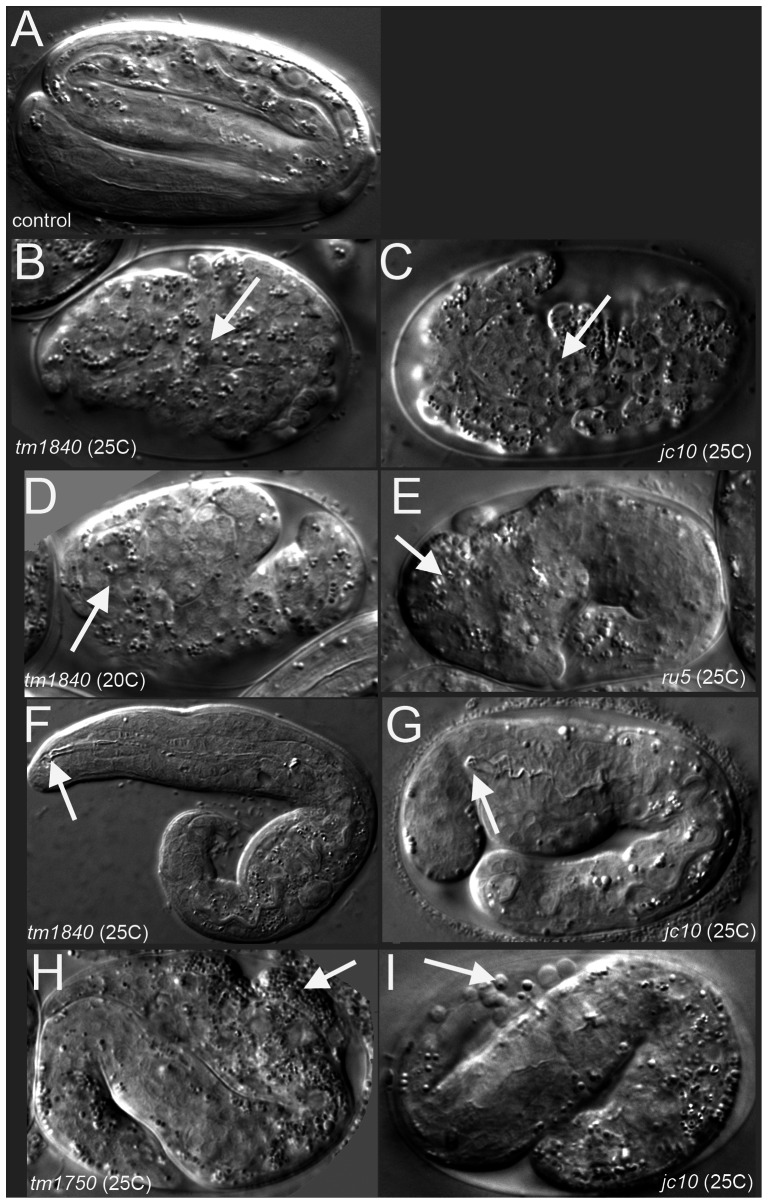
*pmr-1* mutant embryos show a range of terminal phenotypes at the temperatures tested. (A) Control embryos elongate into the vermiform shape just prior to hatching. In contrast, all four mutant alleles of *pmr-1* produce the same range of terminal phenotypes that include B) and C) enclosure defects resulting in full body ruptures (arrow), D) and E) head ruptures (arrow), F) and G) a detached pharynx and body morphogenesis defects (arrow) or H) and I) cells outside the embryo (arrow). Embryos shown include deletion alleles *pmr-1(tm1840)* (B, D, F) and *pmr-1(tm1750)* (H), as well as hypomorphic alleles *pmr-1(jc10)* (C, G, I) and *pmr-1(ru5)* (E). See Table 2 for specific terminal phenotype frequencies for each allele. In all images, anterior is to the left.

Using two independent forward genetic screens designed to identify conditional mutants essential for embryonic enclosure and morphogenesis, we obtained two alleles of the *C. elegans* SPCA1 homolog *pmr-1, jc10 and ru5*. Strains homozygous for either the *jc10* or *ru5* allele produce inviable progeny when grown at 25°C, although these strains show increased embryonic viability at lower temperatures (Table 1). The *tm1840* and *tm1750* alleles [43] show similar temperature sensitivity, although overall embryonic viability is significantly lower at both 15°C and 20°C. For all four alleles examined, *Pmr-1* embryos that die during mid-embryogenesis display a range of terminal phenotypes, including ventral closure defects, head ruptures, morphogenesis defects, and pharynx unattached (Pun) phenotypes (Figure 1; Table 2).

**Table 1:** The severity of the phenotypes in *pmr-1* mutant strains is dependent on allele and temperature.

	Average % Viability of Progeny^1^^,^ 2						Average Brood Size^1^^,^ 2			
Parental Genotype:	15°C		20°C		25°C		15°C		25°C	
N2	100	a	99	a	96	a	210	a	182	a
*tm1840*	61	b	2.9	b	0.0	b	155	b	68	b
*tm1750*	67	b	1.9	b	0.1	b	180	c	119	c
*jc10*	87	c	18	c	0.2	b	152	b	63	b
*ru5*	94	c	12	c	0.2	b	199	c	113	c
*ru5/+*					100	a				
*tm1840/+*					97	a				
*tm1750/+*					95	a				
*jc10/+*					97	a				
*tm1750/ru5*					0.0	b				
*jc10/ru5*					0.6	b				
*N2(RNAi)* 3	92	c								
*tm1840(RNAi)* 3	74	b								
*ru5(RNAi)* 3	93	c								

1 Each strain showed significant differences in brood size or viability when compared at each temperature except N2, which showed no differences in brood size between 15°C and 25°C or in viability between 15°C and 20°C.

2 Letters indicate strains that are not significantly different from each other, but have significant differences from the other strains tested at the indicated temperature. T-test; significant values are p<0.05; n = 7 to 12 for all strains tested. The number of total embryos examined in viability experiments ranged from 341 to 2738.

3 RNAi indicates *pmr-1(dsRNA)* construct. See Materials and methods.

**Table 2:** *pmr-1* alleles are pleiotropic at all temperatures.

		% Terminal Phenotypes^1^			
genotype	T (°C)	Mild	Moderate	Severe	n
*tm1840*	15	39	23	38	44
*tm1840*	20	64	9	27	20
*tm1750*	20	50	15	35	26
*jc10*	20	57	0	43	23
*ru5*	20	62	15	23	32
*tm1840*	25	55	21	24	30
*tm1750*	25	53	28	19	32
*jc10*	25	58	15	27	41
*ru5*	25	51	26	23	37
*tm1750/ru5*	25	66	29	5	41
*jc10/ru5*	25	60	27	13	32

1 No significant differences in frequencies of terminal embryonic lethal phenotypes except for *tm1840* at 15°C, which showed a reduced number of mild phenotypes; X^2^ test p = 0.05. Severe phenotypes are enclosure failures, moderate phenotypes include head and body ruptures, and mild phenotypes include PUN and body morphogenesis defects.

We established that the embryonic lethal phenotypes observed in *jc10* and *ru5* strains are due to mutations in the *pmr-1* gene by a series of criteria. Using conventional and snp-snp mapping strategies, the *jc10* and *ru5* alleles were mapped to a small interval on L.G. I (Figure S1A). Whole genome and conventional sequencing revealed that *pmr-1* was the only gene in the mapped region that carried a mutation in both the *jc10* and *ru5* strains. Complementation analysis showed that *jc10* and *ru5* failed to complement each other and also failed to complement the *pmr-1* deletions *tm1750* and *tm1840*, consistent with the interpretation that all four mutations are allelic (Table 1; data not shown; Figure S1B). Transformation of *ru5* strains with fosmids that contain the *pmr-1* gene (Figure S1B, S1C) rescued the high temperature embryonic lethality. In contrast, we did not observe rescue in strains transformed with any other fosmids or cosmids from the mapped region (Figure S1B, S1C). Taken together, these experiments confirm that the defects in embryogenesis that we observe are due to disruption of *pmr-1* gene activity.

The *pmr-1(tm1840)* deletion allele most likely represents a null, as the deletion eliminates an early exon, found in all of the major identified isoforms (Figure S1B) [33]. Based on strong sequence homology between PMR-1 and SERCA, whose crystal structure has been solved [44], the deletion would eliminate a transmembrane domain and a portion of the E1/E2 ATPase domain, thus rendering the expressed protein non-functional. However, we were unable to confirm whether the *pmr-1(tm1840)* allele is a null. Genetic tests with the dxDf2 deficiency strain were uninformative due to strong dominant phenotypes in the parental strain. Injection of dsRNA did not enhance or diminish the defects in the *pmr-1(tm1840)* strain (Table 1), which is consistent with expectations for a null mutation. However, injection of dsRNA into controls did not phenocopy the *pmr-1(tm1840)* allele, a result consistent with those reported by others (Table 1); [35], [45]. Alternative experiments to test for the null, such as use of the antisera generated against PMR-1 [34], were also ambiguous because of background activity of the antisera (data not shown).

While the *tm1840* allele has the highest mortality at low temperatures, other alleles of *pmr-1* show a range of phenotype severity. The *pmr-1(tm1750)* allele contains a deletion of coding sequence from just one isoform, but the severity of the phenotype, which is similar to *pmr-1(tm1840)*, suggests either the isoform is critical or other isoforms are also disrupted (Table 1 and Table 2). The *pmr-1(jc10)* allele has a premature stop in an exon found in all isoforms (exon 4 of the *pmr-1b* isoform) that would eliminate transmembrane helices and the E1/E2 ATPase domain (Figure S1). The *pmr-1(ru5*) allele is a G142D substitution in a residue conserved from yeast to humans in PMR-1, but also conserved in SERCA (Figure S1) [46]. Based on homology with SERCA, this residue, in a beta strand that forms a portion of the A domain, would likely result in localized disruption of this domain, interfering with key interactions between the A and N domains of the E1/E2 ATPase [44]. Because all four alleles show the same embryonic lethal defects, varying only in the frequency of inviable embryos observed at a given temperature, the simplest interpretation is that they represent reduced or complete loss of function of *pmr-1* gene activity, and are indicative of a key role for *pmr-1* in embryogenesis.

In humans, Hailey-Hailey disease has a dominant inheritance pattern, likely due to haploinsufficiency of the *pmr-1* homolog SPCA1 [10]. In *C. elegans*, analysis of dominance is complicated because we observe both maternal and zygotic rescue for *pmr-1* alleles. *pmr-1(ru5)* hermaphrodites crossed to control males produce live progeny at 25°C, showing zygotic activity is sufficient to rescue the mutant phenotype. F1 hermaphrodites from a cross between *pmr-1* and control strains produce between 96%–100% viable F2 embryos at 25°C, depending on the allele (Table 1). Since 25% of these F2 progeny should be homozygous for the *pmr-1* loss-of-function alleles, these data suggest the maternally provided gene product is sufficient to rescue most of these embryos. However, for *pmr-1(ru5)*, we were able to determine that the inheritance pattern is weakly semi-dominant. Of F2 progeny from a cross between *pmr-1(ru5)* and controls, 39% show a later developmental phenotype at 25°C, including larval lethality (3%), sterility (13%), small broods (3%), and inviable F3 progeny (20%; n = 236). These data show that some individuals heterozygous for *pmr-1* loss-of-function alleles exhibit a phenotype. Although the literature reports that *pmr-1* is expressed in most blastomeres throughout embryonic development [36]–[38], these genetic data suggest the wild type *pmr-1* gene product is essential during a developmental period when both maternal and zygotic gene products are active.

### *pmr-1* is required for embryonic development

During *C. elegans* embryonic development the epidermal (hypodermal) cells that are born on the dorsal surface migrate to the ventral midline, enclosing the body of the embryo. The anterior-most epidermal cells also migrate to enclose the head, ultimately connecting epidermal tissue with the anterior foregut, creating the buccal cavity (Figure 1A) [47]–[49]. *pmr-1* mutant embryos die with variable terminal phenotypes that include failure to enclose the body (Figure 1B, 1C) and head Figure 1D, 1E), as well as a detached pharynx (Pun) (Figure 1F, 1G). *pmr-1* embryos that elongate also show defects in epidermal cell organization (1H, I). Additional phenotypes include reduced brood size and some larval lethality (Table 1). The terminal phenotypes due to *pmr-1* disruption, such as ventral enclosure defects and head enclosure failures, are at least superficially similar to the neural tube closure failures in mice lacking the PMR-1 homolog SPCA, as well as the cell adhesion defects observed in Hailey-Hailey disease patients [19], [23], [24].

When *pmr-1* is disrupted, the frequency of embryonic lethality varies depending on both allele and temperature. Given that all four alleles carry different types of genetic lesions, from point mutation to deletion, it is unlikely that they are temperature sensitive, per se, but rather there is a developmental process in which normal *pmr-1* activity is increasingly important as temperature is increased. All alleles are also pleiotropic, showing a range of embryonic lethal phenotypes (Table 2). The frequency with which a specific terminal phenotype is observed is similar for all alleles, at all temperatures, and independent of whether the temperature shifts occur early or close to the temperature-sensitive period in development, as described below (Table 2). These data are consistent with the hypothesis that *pmr-1* is playing a role in developmental events that affect cells positioned throughout the embryo, resulting in the variable effects.

### PMR-1 is required during gastrulation

**Figure 2:**
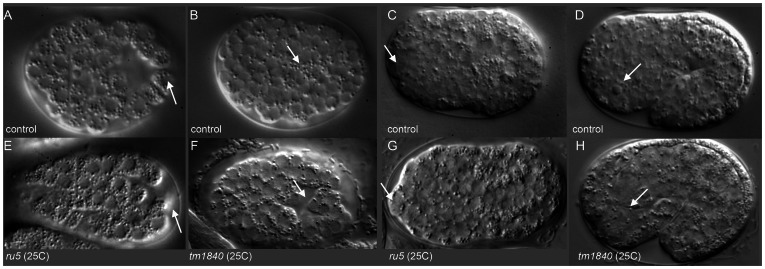
*pmr-1* mutant embryos show differences in the positioning of cells during and after gastrulation. A) In control embryos, the C blastomeres are positioned at the posterior end of the embryo, on the ventral surface, surrounding the gastrulation cleft (arrow). E) In *pmr-1* mutant embryos at the same developmental stage, the C blastomeres are shifted to a more dorsal position, out of the ventral focal plane (arrow). B) In control embryos, the gastrulation cleft closes as the last ventral cell ingresses (arrow). F) In *pmr-1* mutant embryos at the same stage, the gastrulation cleft remains open (arrow). C) In control embryos, anterior cells form a smooth continuum (arrow), but are displaced in *pmr-1* mutant embryos (G, arrow). D) In control embryos, the basement membrane that surrounds the anterior pharynx disappears as these cells migrate to the anterior (arrow, [49]. However, disruption in cell positioning disrupts this process in *pmr-1* mutant embryos (H, arrow), preventing pharyngeal attachment. Genotypes are N2 control (A–E), *pmr-1(tm1840)* (F, H), or *pmr-1(ru5)* (E, G), all grown at 25°C. In all images, anterior is to the left. A–C and E–G are ventral views; D and H are lateral views.

To better understand the defects leading to the Pmr-1 terminal phenotypes, we observed embryos during gastrulation using Nomarski optics. Using this approach, the first observable differences between control and *pmr-1(ru5)* embryos are in the position of the C blastomeres. In control embryos, the C-lineage blastomeres are positioned along the ventral surface at the posterior end of the embryo just prior to ingression (Figure 2A). In *pmr-1(ru5)* mutant embryos at the same developmental stage, these cells occupy a more dorsal position (Figure 2E). At the end of gastrulation, the gastrulation cleft closes in control embryos (Figure 2B). However, in pmr-1(ru5) embryos, the cleft remains open (Figure 2F), reflecting mispositioned ventral neuroblasts. We also saw later differences in cell position, with anterior blastomeres properly positioned in controls (Figure 2C), but altered in pmr-1(ru5) embryos (Figure 2G). Finally, we observed that the basement membrane, which initially surrounds the pharynx but is lost along the anterior most cells of the pharynx in control embryos (Figure 2D) [49], remains in place in *pmr-1(ru5)* embryos (Figure 2H). This analysis suggests the terminal phenotypes we observe are actually caused by problems in cell positioning earlier in embryogenesis.

**Figure 3:**
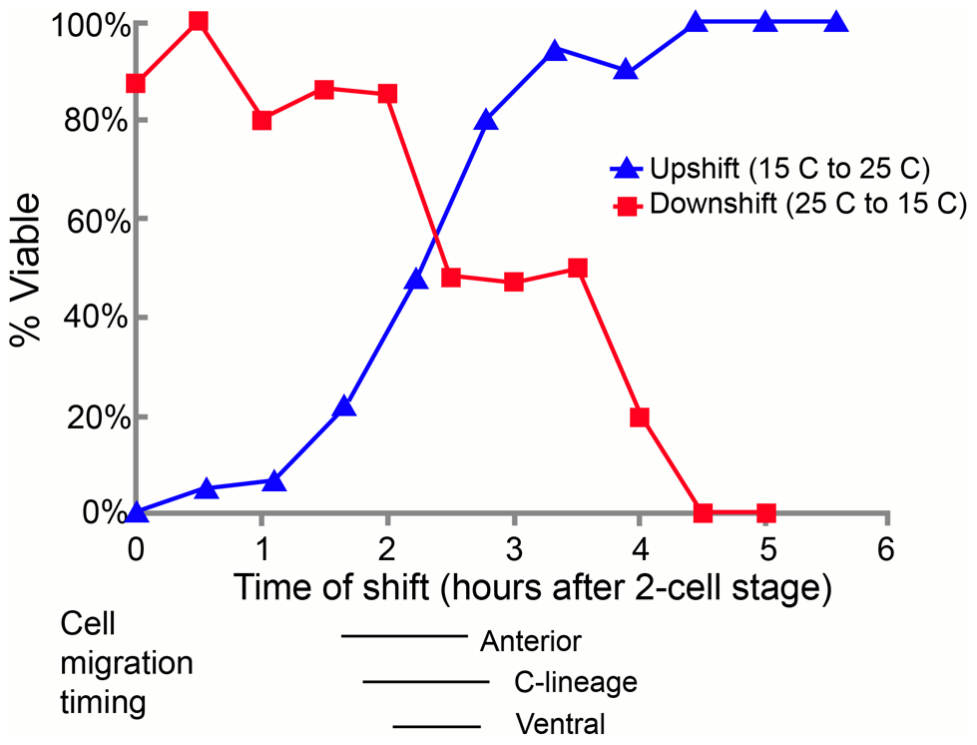
The temperature-sensitive period for *pmr-1(ru5)* embryos is during anterior, C-lineage, and ventral cell migrations. In the upshift experiments, embryos, extracted from gravid adults grown at the permissive temperature (15°C) from the mid-L4 stage, were switched to the restrictive temperature (25°C) at the indicated time. Embryos were then maintained at the restrictive temperature for the duration of embryogenesis and scored “viable” if they thrived past the L1 stage. In the downshift experiments, the same protocol was followed except that the embryos were switched from restrictive temperatures (25°C) to permissive temperatures (15°C) at the indicated times. The lines below the graph correspond to the times when anterior, C-derived, and ventral lineage cell migrations were assayed. All times were normalized to correspond to development at 25°C; n = 5 to 36 embryos at each time point.

To better pinpoint the developmental time point when *pmr-1* activity is required, we took advantage of the temperature sensitive phenotypes of *pmr-1* alleles. Using the *pmr-1(ru5)* allele, which has viable progeny at 15°C but not at 25°C and is less fragile than the *pmr-1* deletion alleles, we performed temperature shift experiments. In reciprocal temperature shifts, we found that embryos grown at the restrictive temperature 2 to 3 hours after the two-cell stage are not viable, while those grown at the permissive temperature during this time period survive (Figure 3). Temperature pulse experiments show a one-hour pulse from the restrictive to permissive temperature can significantly rescue viability only if the pulse occurs between 2 and 3 hours after two-cell stage, consistent with the hypothesis that *pmr-1* is required only during this specific time period (). In contrast, one-hour pulses from 15°C to 25°C are insufficient to cause high lethality (). The temperature-sensitive period for *pmr-1* is during gastrulation, during a period when anterior, c-lineage, and ventral blastomeres migrate (Figure 3; Figure S2), and significantly earlier than the events directly associated with the enclosure failure, Pun, and morphogenetic terminal phenotypes.

### *pmr-1* mutants do not have lineage or cell fate defects

**Figure 4:**
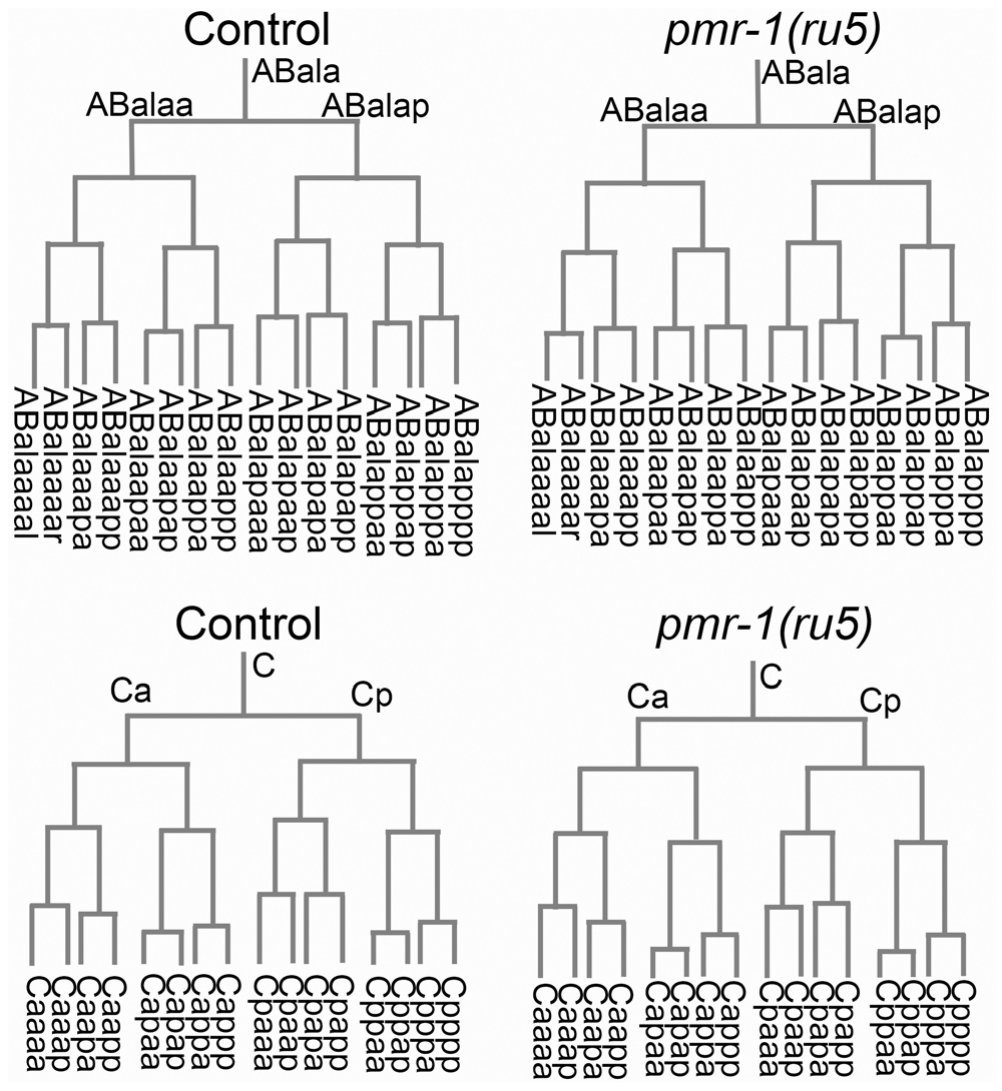
Cell division patterns and timing are normal in *pmr-1(ru5)* embryos. We determined the cell division patterns in control and *pmr-1(ru5)* embryos at 25°C to ∼300-cell stage using 4-D microscopy and cell lineage analysis software (Materials and Methods; n = 6 for each strain). Two representative lineages for control (left) and *pmr-1(ru5)* (right) embryos are shown. We did not observe any differences in cell lineage, cell deaths, or cell division timing in *pmr-1(ru5)* embryos throughout the temperature-sensitive period. Apoptotic cell deaths, which begin at least one cell division after the temperature sensitive period, occurred normally in all lineages examined (n = 2 controls; n = 4 *pmr-1(ru5)* strains).

Given that the temperature-sensitive period is during a stage of development when cells are rapidly dividing and differentiating, we wanted to determine if the defects observed in *pmr-1* embryos were due to changes in cell lineage. In *C. elegans*, the lineage pattern is essentially invariant in control embryos [47]. We took advantage of this to compare the lineage of *pmr-1(ru5)* embryos to controls, from the 2-cell to ∼300-cell stage, using StarryNite software [50]–[53]. Our analysis of *pmr-1(ru5)* embryos indicates that the cell division timing and pattern for all lineages is indistinguishable from controls (Figure 4). We also found that the correct number of cells underwent programmed cell death with normal timing, beginning one to two divisions after the temperature-sensitive period (data not shown). These data indicate that *pmr-1* embryos are not dying because of premature cell death or general defects in cell division timing or patterns.

Although lineages appeared normal in *pmr-1(ru5)* embryos, we wanted to ascertain whether the cell fate was also normal. To address this question, we examined the expression patterns of GFP reporter fusion constructs that are expressed with specific timing, in a position-dependent manner within the embryo, or in specific tissues. Reporters included those expressed in the nervous system, hypodermis, muscle, intestine, and pharynx, some of which are derived from multiple lineages. We also included reporters, such as *ceh-13, ceh-16* and *vab-7*, which are expressed in a position-dependent manner in multiple tissues and lineages [54]–[57]. In comparisons between control and *pmr-1(ru5)* embryos, the expression patterns of all of these reporter constructs were identical with only a few exceptions (Table 3; Figure S3, S4). These exceptions included differences in the position, but not in the number of cells expressing epidermal, neuroblast, and muscle markers. The other exception involved the expression of *vab-7*/even-skipped [54], which showed modest, but statistically significant delays in gene expression in C-lineage muscle and epidermal cells (Table 3; Figure S3). While we cannot rule out subtle effects on cell fate or changes in the fate of a small subset of cells, this analysis indicates that all major lineages and tissues are differentiating properly in *pmr-1(ru5)* embryos grown under restrictive conditions.

**Table 3:** *pmr-1(ru5ts)* strains properly express cell fate and cell polarity markers.

Marker	Expression pattern	Stages examined	Observed Pattern in *pmr-1(ru5)* embryos^3^	Parental Strain; References
*AJM-1*	Adhesion junctions of hypodermis, pharynx, gut	Enclosure and comma	Normal timing, cell number and localization; changes in position of V3-V5^1^.	SU93; [99]
*vab-7*	C-derived hypodermis and body wall muscles.	3 hours after 2-cell	Delayed expression; altered organization^2^.	This study. [54]
*pha-4*	Pharynx	Enclosure and comma	Normal timing, cell number and position^1^.	SM467 and SM469; [49]
*hlh-1*	Body wall muscle	3 hours after 2-cell	Normal timing and cell number; cells mispositioned^1^.	PD7963; [100], [101]
*ceh-16*	Seam hypodermis	Gastrulation, enclosure comma	Normal timing and cell number. Change in position of V3–V5 seam cells^1^.	SU324; [56], [102]
*ceh-13*	A, D, E and MS lineages; anterior body wall muscle and hypodermis	3 hours after 2-cell	Normal timing and cell number^1^.	FR317; [103]
KAL-1	AB-derived neuroblasts	Enclosure	Normal timing of expression; cells mispositioned.	OH904; [104]
PLX-2	Ventral neuroblasts	Enclosure	Normal timing of expression; cells mispositioned.	SU272; [105]
SMA-1	Apical membrane of hypodermis, pharynx, gut	Enclosure and comma	Normal expression and localization pattern.	Antisera, [62]
NID-1	Basement membrane of muscle; around pharynx and gut	Comma and 1.5× stage	Normal localization pattern.	Antisera; [63]
MEL-11	Apical membrane of hypodermis	Comma and 1.5× stage	Normal localization pattern.	Antisera; [68]
VAB-9	Apical membrane of hypodermis	Comma and 1.5× stage	Normal localization pattern.	Antisera; [65]
PAR-6	Apical membranes of pharynx and intestine	Comma and 1.5× stage	Normal localization pattern.	JJ1579; [61], [106]

1 A normal number of cells expressing marker. P>0.05.

2 Expression of *vab-7*::GFP 3 hours after 2-cell stage was significantly reduced. P<0.05; n = 24 (control) and 18 (*ru5*).

3 See Figure S3 and Figure S4 for images of expression patterns.

### Pmr-1 embryos have defects in cell migration

**Figure 5:**
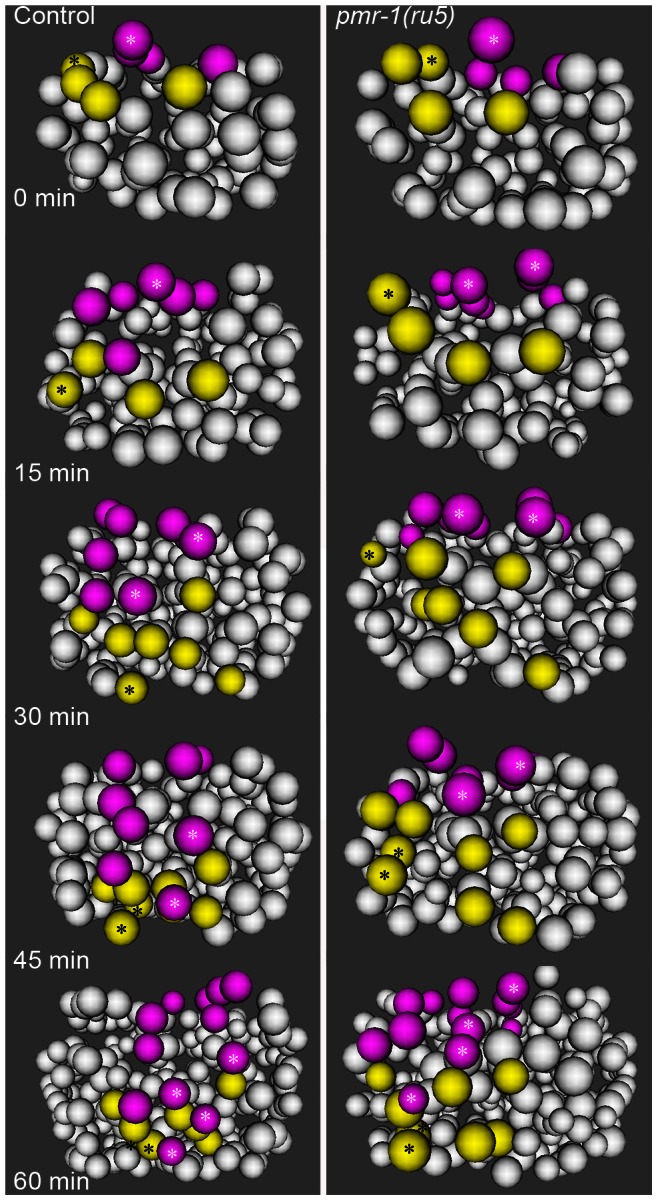
Cell migration defects in the C lineage cells of *pmr-1(ru5)* embryos. In control embryos (left panels), the C lineage muscle (yellow) and hypodermal (magenta) precursors migrate from dorsal to ventral positions. The migration rates of control embryos are significantly faster than in *pmr-1(ru5)* embryos (right panels). The muscle cell precursor Capa and its descendants (yellow; *) and the hypodermal cell precursor Cpap, and its descendants (magenta; *) show the migration and division of a single cell lineage in controls and *pmr-1(ru5)* mutant embryos. Average rates of migration of these cells for *pmr-1(ru5)* embryos are 55% of controls for Capa and 40% for Cpap (n = 6 for each strain; p<0.05). Each panel represents a 15-minute interval, starting at 1.8 hours (∼97 cells) after the 2-cell stage at 25°C, which corresponds with the temperature-sensitive period. Posterior view is shown, with dorsal at top.

The observed differences in the position and timing of the *vab-7* reporter, as well as the Nomarski imaging analysis (Figure 3; Table 3), suggested that *pmr-1* mutants may have defects in cell migration. Since cells that express *vab-7* are derived from the C lineage [54], we looked more closely at cell migration in C-derived blastomeres. In control embryos, the C-lineage blastomeres migrate from a dorsal position to a more ventral one along the posterior surface of the embryo. Muscle precursors, which have a more ventral starting position and migrate ahead of the hypodermal precursors, eventually ingress into the embryo at a posterior/ventral position (Figure 5) [58], [59]. When we compared control and *pmr-1(ru5)* embryos, we saw no differences in the timing of C-blastomere ingressions. Similarly, the timing of E, P4, MS, and D-derived blastomere ingressions were similar in control and *pmr-1(ru5ts)* embryos (n = 6 for each strain; t-test; p>0.05). This analysis shows that *pmr-1* does not appear to be playing an essential role in cell ingression during *C. elegans* gastrulation.

In contrast, *pmr-1* does have a critical role in the migration of cells along the surface of the embryo. Using 4-dimensional representations of embryos created with Acetree, we measured the migration rates of C blastomeres along the posterior surface of the embryo (Materials and methods). In *pmr-1(ru5)* embryos, the rates of migration of both muscle and hypodermal cell precursors were significantly slower, at 55% and 40% of controls, respectively (Figure 5; t-test; p<0.05). Given that these cell migrations occur during the temperature sensitive period for *pmr-1* gene activity, this analysis is consistent with the interpretation that *pmr-1* plays an important role in cell migration during *C. elegans* embryonic development.

**Figure 6:**
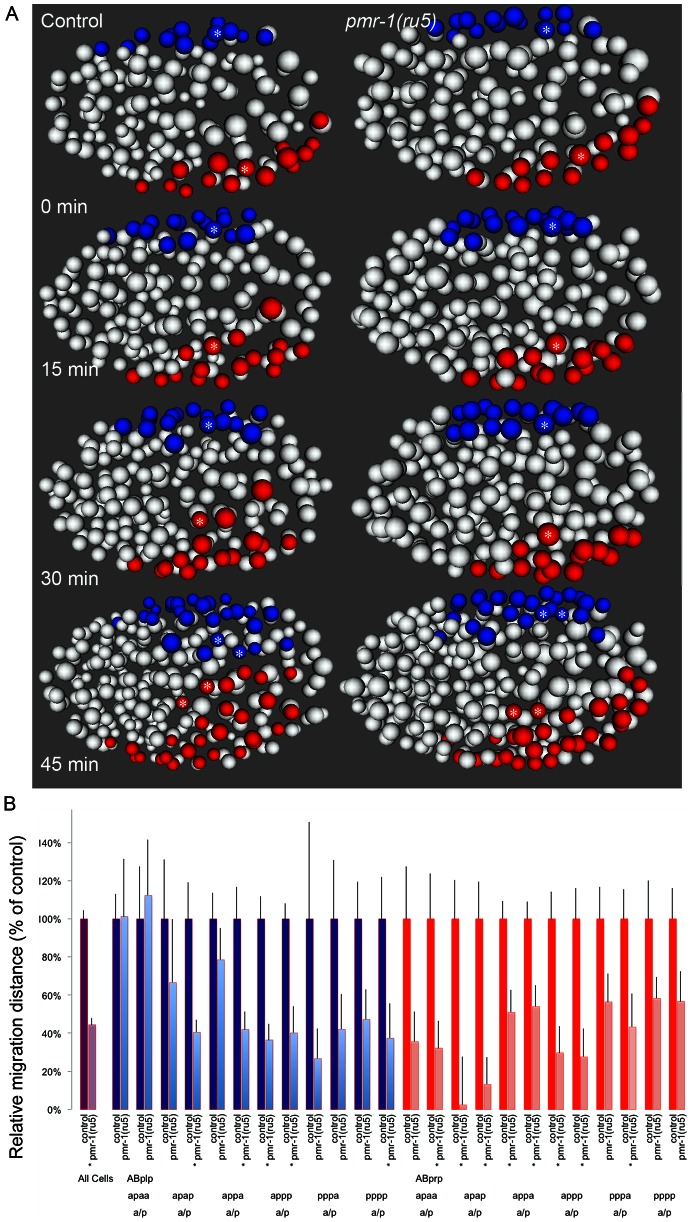
Cell migration defects in ventral cells of *pmr-1(ru5)* embryos. A. Ventral ABp-derived blastomeres migrate from the left (ABplp; blue) or right (ABprp; red) side of the embryo toward the ventral mid-line in control embryos (left panels). In *pmr-1(ru5)* embryos (right panels), the migration of these blastomeres is significantly reduced. For example, the rectal and interneuron precursor AB.plp appp (Blue; *) and the ring interneuron and ventral cord neuron precursor AB.prp appa (Red; *) migrate further in control than in *pmr-1(ru5)* embryos. Each panel represents a 15-minute interval, starting at 2 hours (∼162 cells) after the 2-cell stage at 25°C, which corresponds to the temperature-sensitive period. Ventral view is shown, with anterior to the left. B. In comparisons of average migration distance for ABprp (red) and ABplp (blue) blastomeres, cells migrated significantly farther in controls (dark boxes) than in *pmr-1(ru5)* (light boxes), with significant reductions in migration distance in at least one daughter in 8 of the 12 cells examined. The migration rates in *pmr-1(ru5)* embryos were 44% of those in controls, when all ventral blastomere migration values were compared (purple). T-test, * indicates p<0.05. Specific lineages tested are indicated. Measurements were taken starting at 2 hours after the 2-cell stage at 25°C (∼162 cells), and again 45 minutes later.

Given the variability of the *pmr-1* terminal phenotypes, we examined several other lineages to determine if other cells exhibited cell migration defects. Terminal phenotype and gene expression analysis suggested that many cells are properly positioned in the embryo, so we focused our attention on cells that migrate during the temperature-sensitive period and whose migration defects might lead to enclosure failures and Pun phenotypes. In control embryos, the ventral neuroblasts derived from ABprp and ABplp lineages migrate from lateral to central positions along the ventral surface of the embryo, closing the gastrulation cleft (Figure 6A; [47], [58]. Hypodermal cells then crawl over these cells during enclosure [47], [48]. Analysis of *pmr-1(ru5)* embryos showed that these ventral cells are properly positioned at the beginning of the temperature-sensitive period, but many move a shorter distance than controls (Figure 6A), in some cases failing to close the gastrulation cleft, leading to later enclosure failures. The migration distances of lineages examined varied widely, and some cells migrated distances similar to controls. However, the majority of cells examined showed significant reduction in cell migration, on average 44% of controls in *pmr-1(ru5)* embryos (Figure 6B; T-test; p<0.05). These results show that *pmr-1* is playing a key role in cell migration in cells that give rise to ventral neuroblasts, as well as epithelial cells.

**Figure 7:**
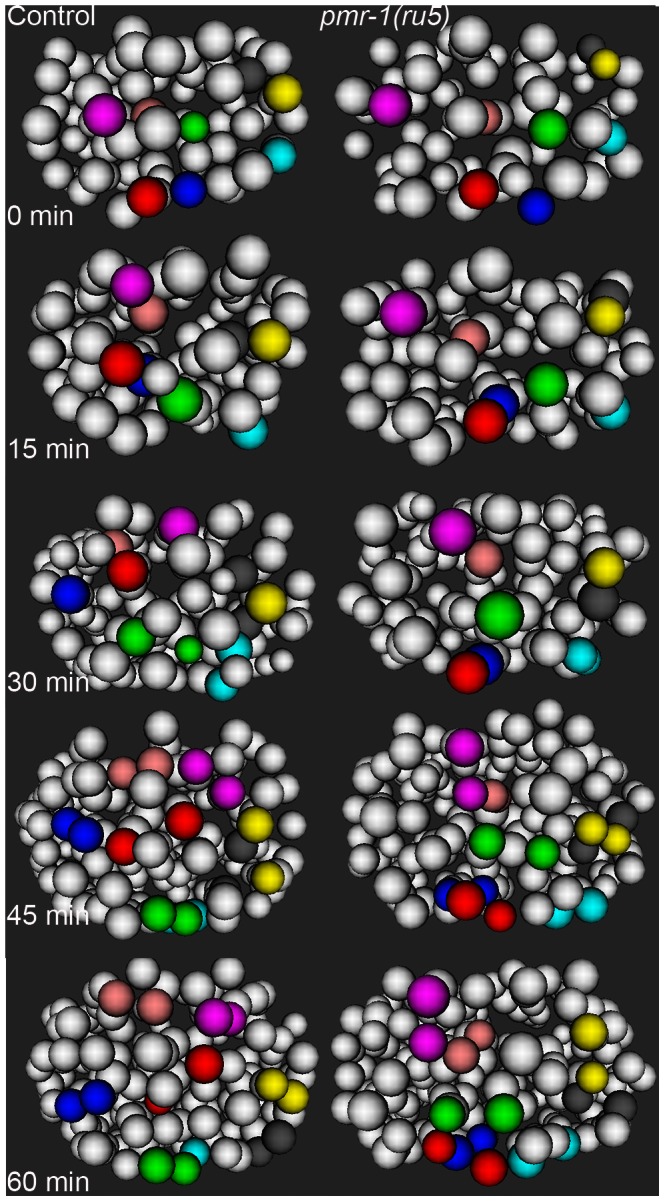
Cell migration defects in the anterior-most cells of *pmr-1(ru5)* embryos at 25°C. The anterior cells of control embryos (left panels) undergo distinct and reproducible cell rearrangements during gastrulation. CANL, labial, and ring ganglia precursors ABalapaa (magenta) and ABalaapa (blue) cross the left/right mid-line, labial and ring ganglia precursors ABalpppa (yellow) and ABalppap (gray) cross the dorsal/ventral mid-line and pharyngeal precursor ABalpapp (cyan) migrates from the left to a ventral position. Other cells, such as ring ganglia precursor ABalaaaa (red), migrate from peripheral to central positions or vice versa, as with pharyngeal precursor ABalpaaa (green) and labial/ring ganglia/CANL precursor ABalappa (pink). In a *pmr-1(ru5)* embryo (right panels), many of these anterior cells migrate shorter distances, migrate in the wrong direction, or fail to change directions, resulting in cells that are mis-positioned. While not every cell showed a migration defect in each embryo, measurements of the Cartesian coordinates for all of these 8 cells except ABalpppa (yellow), as well as three others not labeled above (labial and ring ganglia precursors ABalaaap, ABalpppp, and ABalppaa) showed significant positional differences in *pmr-1(ru5)* embryos (n = 6) compared to control embryos (n = 6; p<0.05). Each panel represents a 15-minute interval, starting at 1.6 hours (∼88 cells) after 2-cell stage at 25°C, which corresponds to the temperature-sensitive period. Anterior view is shown, with dorsal (top) and embryo right (left). Cell fate information from [97], [98].

In the anterior of control embryos, cells derived from the ABala and ABalp lineages, many of which ultimately differentiate into epithelial or nervous system cells, undergo dynamic migration patterns, with individual blastomeres crossing the dorsal-ventral or left-right midlines during embryogenesis. Some anterior blastomeres also make specific turns and ingress to form the anterior pharynx (Figure 7; [47], [58], [60]. In *pmr-1(ru5)* embryos, many anterior cells show migration defects. While some cells migrated normally in a subset of the *pmr-1(ru5)* embryos examined, many cells migrated shorter distances, in the wrong direction, or failed to make key turns, resulting in cells that were improperly positioned (Figure 7; t-test; p<0.05). Since these cells give rise to arcade cells, hypodermis, and ring ganglia, their failure to migrate properly during the temperature-sensitive period can readily account for the terminal phenotypes we observe in *pmr-1(ru*5) embryos. Taken together, these results show that *pmr-1* plays a key role in cell migration and positioning during gastrulation.

### *pmr-1* mutants do not show disruptions in cell polarity, adhesion, or basement membrane structures

In Hailey-Hailey disease, cells show defects in cell attachment structures [23], [24]. The defects in cell migration we observe in *pmr-1* mutants could be due to changes in cell adhesion, as well as in other cellular processes including cell polarity or establishment of the basement membrane. Our analysis of gene expression suggests that each of these processes is relatively normal in *pmr-1(ru5ts)* embryos. PAR-6 is an apically localized protein that plays a key role in cell polarity in a number of developmental processes [61]. SMA-1 is an actin-binding protein that localizes to the apical membrane of polarized epithelial cells [62]. NID-1 is a basement membrane protein expressed during embryonic development and *nid-1(cg119)* mutants have Pun phenotypes [63], [64]. AJM-1, MEL-11, and VAB-9 localize to apical adhesion complexes [65]–[68]. Each of these proteins is expressed in the correct cells and shows normal subcellular localization in *pmr-1(ru5ts)* embryos (Table 3; Figure S4). The localization is normal even in hypodermal cells that show positioning defects (Figure S4M-S4R). Similarly, KAL-1 and PLX-2, proteins that are expressed in ventral neuroblasts and play key roles in hypodermal cell migration and enclosure [69], [70], appear to be properly expressed in *pmr-1(ru5*) embryos (Figure S3I–S3L). While we cannot rule out subtle changes in protein localization and gene expression, these data indicate that the defects we observe in *pmr-1* mutant embryos are not due to general defects in cell polarity, cell adhesion, or expression of basement membrane proteins.

### Altering calcium channel activity can modulate *pmr-1* mutant phenotypes

Given the conserved molecular roles of PMR-1/SPCA, we asked whether perturbations in Ca^2+^ signaling were chiefly responsible for the *pmr-1* mutant phenotypes. To address this question, we altered the activity of either the ITR-1/IP3R or UNC-68/RyR calcium channels, reasoning that if calcium levels were critical for normal cell migration, changes in the activities of these receptors would suppress or enhance the *pmr-1* embryonic lethal phenotypes. We made strains carrying *pmr-1* mutations and *itr-1(sy327*), a gain-of-function allele that is thought to increase the affinity for IP3 [28], [71]. At 20C, the *itr-1(sy327*) strain has slightly reduced embryonic viability (91%) compared to controls while the *pmr-1(tm1840)* strain has 9% embryonic viability. The *pmr-1(tm1840);itr-1(sy327)* double mutant shows significant improvements in viability compared to the *pmr-1(tm1840)* single mutant (42%; Table 4). This affect is not allele specific, as we observed similar suppression of phenotypes for the *pmr-1(jc10)*, *pmr-1(ru5)*, and *pmr-1(tm1750)* lines when placed in an *itr-1(sy327)* background (Table 4). These data show *itr-1* phenotypes are epistatic to *pmr-1*. We also made double mutants carrying *pmr-*1 alleles and *itr-1(jc5)*, a loss-of-function allele [27]. While we were unable to generate double mutants with *pmr-1(tm1840)* or *pmr-1(tm1750)*, suggesting the combination is lethal (data not shown, V.P., R.W. and R.M.), the *pmr-1(ru5); itr-1(jc5)* strain showed enhanced lethality compared to the *pmr-1(ru5)* strain alone (Table 4), suggesting the two genes act in parallel pathways during this stage of development. In contrast, depletion of *unc-68* using RNAi significantly suppressed the embryonic lethality of *pmr-1(ru5)* strains (Table 4), indicating *unc-68* acts in opposition to *pmr-1* in a role very different from that of *itr-1*. Taken together, these data show that the embryonic lethality we observe in *pmr-1* mutant embryos can be affected by changes in the activity of calcium channels. While this type of analysis does not allow us to rule out other possibilities, it provides strong support for the hypothesis that *pmr-1* is acting through its role in Ca^2+^ signaling to affect cell migration.

**Table 4:** Genetic interactions with *pmr-1*.

Genotype	Temp	% viable^1^		n
*pmr-1(tm1840)*	20C	9	a	743
*pmr-1(tm1750)*	20C	7	a	665
*itr-1(sy327 gf)*	20C	91	b	886
*pmr-1(tm1840); itr-1(sy327)*	20C	42	c	487
*pmr-1(tm1750); itr-1(sy327)*	20C	34	c	517
*pmr-1(ru5)*	24C	8	a	565
*pmr-1(jc10)*	24C	4	a	289
*itr-1(sy327 gf)*	24C	90	b	297
*pmr-1(ru5); itr-1(sy327)*	24C	22	c	1434
*pmr-1(jc10); itr-1(sy327)*	24C	21	c	261
*pmr-1(ru5)*	20C	26	a	603
*itr-1(jc5 cs lf)*	20C	60	b	567
*pmr-1(ru5); itr-1(jc5)*	20C	14	c	1000
*N2; L4440 RNAi control*	20C	97	a	1067
*N2; unc-68(RNAi)*	20C	99	a	1249
*pmr-1(ru5); L4440 RNAi control*	20C	10	b	2027
*pmr-1(ru5); unc-68(RNAi)*	20C	59	c	1755

1 Letters indicate strains that are not significantly different from each other, but have significant differences from the other strains tested in each cluster. T-test; significant values are p<0.05; n = 10 to 49 hermaphrodites produced progeny for all strains tested.

## Discussion

Our analysis of PMR-1/SPCA1 in *C. elegans* has revealed a novel and essential role in cell migration for this highly conserved gene [1], [9]. Using a forward genetic screen designed to look for genes required for normal epidermal cell migration and adhesion, we identified mutations in the *C. elegans* SPCA1 gene *pmr-1*. Strains homozygous for *pmr-1* show temperature-sensitive embryonic lethality, with terminal phenotypes that include ventral enclosure failures, head ruptures, and morphogenetic phenotypes including a detached pharynx (Pun) phenotype. Phenotype and temperature shift analysis indicates that the *pmr-1* gene plays a crucial role in a specific period of embryonic development, during gastrulation. Cell fate specification, cell division patterns, apoptosis, and cell ingression during gastrulation are all similar to control embryos. However, the migrations of specific subsets of cells, along the posterior, anterior, and ventral surfaces of the embryo, are defective in *pmr-1* mutant embryos. The specificity of the phenotype, coupled with the temperature shift analysis, indicate that PMR-1/SPCA plays an essential role in a specific set of cell migrations during gastrulation in the developing *C. elegans* embryo.

Given the broad expression pattern for *pmr-1* in all cells, coupled with the proposed housekeeping functions one would expect for a calcium transporter [1], [9], the specific timing and proposed role for PMR-1 in ectodermal cell migration is unexpected. Studies examining the expression of *pmr-1* indicate that PMR-1 protein localizes to the Golgi and that the gene product is present in the germ line and widely expressed in cells throughout embryogenesis, including during gastrulation [33]–[38]. This expression pattern, which is consistent with our genetic analysis that maternally or zygotically provided gene product is sufficient to rescue the Pmr-1 mutant phenotype, would suggest a broad role for the gene product. Yet our temperature shift and pulse analysis indicates requirements for PMR-1 can be bypassed during other stages of embryogenesis. The phenotype analysis identifies a very specific role in the migration of anterior, C-lineage, and ventral cells along the surface of the embryo during gastrulation, but with no discernable effects on other cell migration events such as ingression or ventral closure, nor on any other developmental pathway that might alter cell fate, division patterns, or cell polarity. The best explanation for these data is that PMR-1 may act redundantly in some aspects of embryogenesis, but plays a more critical or specific role in the cell migrations along the embryo surface that follow cell ingression, a markedly different developmental process [58], [60].

PMR-1/SPCA is part of a network of proteins that regulate calcium levels in the cell cytoplasm and calcium stores in the secretory pathway. Models for modulating cellular calcium levels start with a signal transduced through PLC and IP3 triggering release of Ca^2+^ into the cytoplasm from secretory stores through ITR-1/IP3 and UNC-68/RyR. The calcium ATPases SCA-1/SERCA and PMR-1/SPCA, located predominantly in the ER and Golgi, respectively, then pump Ca^2+^ back out of the cytoplasm into these organelles [7], [26]. A simple model would predict that reduced activity of a transporter acting in one direction is suppressed by reduced activity of channel acting in the opposite direction. This is the result we observe for interactions between *pmr-1* and *unc-68*, where *pmr-1(ru5)* strains showed reduced lethality in an *unc-68(RNAi)* background (Table 4). In contrast, strains carrying *pmr-1* and *itr-1(jc5)* loss-of-function alleles showed enhanced lethality at semi-permissive conditions while strains carrying the *itr-1(sy327)* gain-of-function allele showed significant suppression of *pmr-1(ru5)* embryonic lethality. This gene interaction analysis suggests that *itr-1* and *pmr-1* act in parallel pathways during embryonic development and that enhanced sensitivity of *itr-1* to IP3 signaling in the *itr-1(sy32y7)* mutant [72] can help bypass the requirement for *pmr-1*. While these interpretations reflect the genetic analysis, they are puzzling because ITR-1 and PMR-1 act in opposite directions with respect to cytosolic calcium. Similarly, UNC-68 does not appear to have any developmental roles nor does it appear to be expressed at high levels in the embryo until later stages [7], [29]–[32].

A speculative model to explain the genetic interaction data we observe is that these proteins may play some redundant and some non-redundant roles in regulating calcium levels in the cell, due in part to the polarity of the secretory pathway. One would predict that when PMR-1 activity is reduced, the pool of calcium in secretory stores is also modestly reduced and the calcium levels in the cytoplasm might be elevated [34]. Reduction of UNC-68 or ITR-1 calcium release would, in theory, restore overall secretory pool levels. However, the calcium flicker model for cell migration predicts that calcium must be supplied in localized bursts at the leading edge of the cell [5], [6]. In a *pmr-1* mutant, the pool of available calcium would be disproportionately reduced in the Golgi, the secretory organelle closest to the leading edge. If release is, as predicted, strongly dependent on ITR-1 [5], [6], an *itr-1* loss-of-function mutant would further diminish calcium release, making the phenotype worse, while the *itr-1(sy327)* gain-of-function mutation would release calcium at lower levels of signal [28], [71], suppressing the phenotype. A second possibility to explain the suppressor data reflects the distinct mechanisms by which the channels are activated. While both the UNC-68/RYR and ITR-1/IP3R channels are sensitive to changes in cytosolic calcium, only the ITR-1/IP3R channels responds to an IP3 ligand [73], [74]. Reduced activity of the ITR-1/IP3R channel would make the secretory pathway insensitive to IP3 signaling, which when coupled with reduced stores would make the phenotype worse. Increasing IP3R sensitivity might compensate for altered cytosolic calcium, making the situation better, as observed. Finally, since the regulation of calcium signaling and transport is highly interdependent, with channels and targets acutely sensitive to minor changes in calcium concentration [1], [72]–[74], single loss-of-function mutations may result in some compensatory changes in the signaling system that cannot occur if two systems are defective.

While it is tempting to assume that disruption of *pmr-1* has a direct effect on calcium-mediated cell signaling, disruptions in the gene might instead effect other calcium-dependent processes required for cell migration. The embryonic lethal phenotypes in Pmr-1 strains were observed at all the temperatures assayed, but they were more severe at high temperatures for all four alleles tested. Although our experiments were in the normal temperature range for *C. elegans* growth [75] and we saw no evidence of necrosis in *pmr-1* mutant embryos, recent research indicates that *pmr-1* is important for survival of heat shock, acting to prevent necrosis [42]. It is possible that the migrating cells of the embryo are particularly sensitive to changes in the activity of this stress resistance pathway. Alternatively, the cytoskeletal and membrane dynamics of migrating cells may simply be more sensitive to temperature than other developmental processes, or the timing is more precise, such that changes in *pmr-1* activity have a stronger impact. Another possibility is that the changes in *pmr-1* activity alter calcium-dependent processing of specific proteins in the Golgi. No matter the precise mechanism by which changes in calcium dynamics alter cell migration, our identification of a role for PMR-1, acting with ITR-1/IP3R and UNC-68/RyR, confirms earlier work that ITR-1 plays an important role in cell migration [27], [28] and identifies new genes important for normal cell migration in the *C. elegans* embryo.

The phenotypes we observe in *C. elegans pmr-1* mutants have at least some superficial similarities to those observed in vertebrates with defects in the SPCA1 gene, including humans with Hailey-Hailey disease. In both *C. elegans* and in vertebrates, we observe semi-dominant phenotypes and the severity of the observed phenotypes are influenced by environmental stresses. The terminal phenotypes, including apparent loss of cell adhesion in the epidermis of humans with Hailey-Hailey or failures to close the rostral neural tube in mice embryos homozygous for SPCA loss-of-function alleles [12]–[16], [19], [23], [24] also look similar to the enclosure failures and head ruptures we observe in *C. elegans* embryos.

One major difference between the worm *pmr-1* mutant phenotype and Hailey-Hailey disease patients is the nature of defects in cell adhesion. In Hailey-Hailey disease, symptoms include acantholysis of keratinocytes, marked by keratin dissociation from desmosomes [23], [24]. Our analysis of *pmr-1* mutants suggests that the gene does not play a direct role in cell adhesion. Proteins that localize to adhesion complexes, such as MEL-11, AJM-1, and VAB-9, look normal in *pmr-1* mutant embryos and epistasis analysis indicates that *vab-9* and *pmr-1* are in different pathways (unpublished data, J. Simske). While intermediate filament and desmosomal structures have not yet been carefully examined in *pmr-1* mutants, we do not suspect an association. While *pmr-1* is essential during gastrulation, analysis by others indicates genes that encode intermediate filament and attachment structures in *C. elegans*, such as IFA-1, IFA-2/MUA-6, IFA-3, IFA-4, IFB-1, VAB-10, MUP-4, and LET-805, assemble later in development, playing key roles in the embryonic elongation that follows ventral closure [76]–[83]. This analysis strongly suggests intermediate filaments are unlikely to be directly involved in the PMR-1-mediated cell migration, although it is possible PMR-1 may play a later, non-essential role in maintaining these attachment structures. Similarly, we did not see elevated levels of cell death in *pmr-*1 mutants, as reported for the mice knockouts [19]. These data suggest that there may be differences between SPCA function in *C. elegans* and in vertebrate systems, perhaps due to fundamental differences in ectodermal cell structure. Alternatively, the terminal phenotypes observed in vertebrates could reflect earlier defects.

Given our results in *C. elegans*, as well as the strong sequence conservation of the gene, it is tempting to speculate that the SPCA's have cell migration roles in vertebrates. The relative simplicity of *C. elegans* as model system, as well as powerful new lineaging tools [50]–[53], has allowed us to distinguish between the terminal phenotype and the actual defects in cell migration found in *pmr-1* mutants. In humans, keratinocytes differentiate in response to changes in extracellular calcium, migrating from the stratum basale into upper layers of the epidermis as they differentiate [84]. It is conceivable that in Hailey-Hailey patients, stress-damaged cells are replaced inefficiently because of a defect in cell migration, caused by altered calcium levels, leading to the observed lesions and keratinocyte defects [23], [24]. Similarly, the rostral neural tube closure failings in SPCA mouse knockouts [19] could be due to defects in migration of specific subsets of ectodermal cells during this stage of development.

In summary, we have shown that the PMR-1/SPCA1 Ca^2+^/Mn^2+^ ATPase plays a key role in cell migration during *C. elegans* embryonic development. Strains carrying mutant *pmr-1* show defects in migration of cells along the surface of the embryo following the ingression of cells during gastrulation. The cell migration defects are likely caused by changes in intracellular calcium levels, as they can be enhanced or suppressed by changes in activity of the ITR-1/IP3R or UNC-68/RyR calcium channels. This analysis reveals a new role for calcium signaling in the migration of specific blastomeres during *C. elegans* development. It also reveals a new role in cell migration for the SPCA1 family of genes.

## Materials and Methods

### Growth conditions, strains, and alleles

Strains were grown and maintained under standard conditions [85]. Wild type strain N2 was used as a control. The *ru5* and *jc10* alleles of *pmr-1* were identified in two independent embryonic conditional embryonic lethal screens using the mutagen EMS [85]; J. Simske). The *tm1840* and *tm1750* alleles were produced in the National Bioresource Project (kindly provided by S. Mitani; [43]. All *pmr-1* mutant strains, which were maintained at 15°C, were backcrossed to the N2 strain at least 3 times. The deletions in *tm1840* and *tm1750* were confirmed using PCR analysis. PCR mapping lines CB4856, RW700, deficiency lines CB2775 *eDf9/eDf24* I, CB2770 *eDf4/eDf24* I, CB2769 *eDf3/eDf24* I, KR2838 *hDf17/hIn1*[*unc-54*(h1040)], JK1542 *ces-1(n703*) qDf7/*dpy-5(e61) srf-2(yj262) unc-75(e950)* I, SL536 dxDf2/*spe-9(eb19) unc-101(m1)* I, and conventional mapping lines MT465 *dpy-5 (e61)*I, *bli-2(e768)*II, *unc-32(e189*)III, MT464 *unc-5(e53)*IV, *dpy-11(e224)*V, *lon-2(e678)*X; DR210 *dpy-5(e61), daf-16(m26), unc-75(e950) I*, CB2010 *unc-54(e675), dpy-5(e61)*I, JK228 *glp-4(bn2), unc-54(e675) I* were used in mapping experiments. SU180 *itr-1(jc5) jcIs1* IV, PS2368 *itr-1(sy327) unc-24(e138)* IV and QQ101 *vab-9(ju6) II* strains were used for double mutant analysis. For analysis using GFP fusions, F2 progeny exhibiting the Pmr-1 phenotype and carrying the appropriate markers were selected from crosses between *pmr-1(ru5)* males and the following strains: RW10026 *unc-119(ed3); stIs10026,* SM467 *pIs7*; *rol-6(su1006)*, SM469 *pIs6*, SU93 *jcIs1*, PD7963 *ccIs7963*, SU324 *jcIs26*, FR317 *swIs1*, OH904 *otIs33*, SU272 *jcIs1*, *evIs136*, and JJ1579 *zuIs77*. Strains expressing vab-7::GFP were generated by microparticle bombardment of pJA64 and pDPMM016b into a *pmr-1(ru5)I; unc-119(ed3)III* strain [86] and backcrossed to wild type to generate controls.

### Mapping, complementation, and genome-sequencing experiments

The *ru5* and *jc10* alleles were mapped using conventional and deficiency mapping strategies [87]. The position of the *ru5* allele was further refined using PCR-based and snp-snp mapping techniques [88]–[90]. Genomic DNA was extracted from the *ru5* strain using standard methods and the whole genome was sequenced using Illumina sequencing and MAQGene, as described [91]. Alterations in candidate genes were confirmed in *ru5* using PCR amplification followed by subcloning (Strataclone, Agilent Technologies) and conventional sequencing (U. of Iowa Carver Center, U of Wisconsin, and Fred Hutchinson DNA sequencing facilities). The coding regions of these candidate genes were then sequenced in *jc10*. In complementation analysis, *ru5* males were crossed to *jc10*, *tm1840*, or *tm1750* hermaphrodites and the F1 progeny scored for embryonic lethality at 25°C. For complementation rescue analysis, *ru5* young adults were transformed by injection of 25–100 µg/ml of a fosmid or cosmid covering each of the candidate genes, along with the co-transformation marker pRF4 [92], [93]. Rol Progeny were assayed for survivorship at 25°C for each injected construct.

### Mutant and RNAi viability and brood size analysis

To determine rates of viability, L4 hermaphrodites were shifted from 15°C to the indicated temperature. Eggs from single hermaphrodites were counted and the number of resultant late larva or adult progeny scored. In brood size experiments, single L4 hermaphrodites were shifted to the indicated temperature and total broods quantified as previously described [94]. Both brood size and viability data were compared using t-tests. Terminal phenotypes were scored during mid-to-late embryogenesis. Embryos that failed to enclose were classified as “severe”, those with head or later body ruptures as “moderate”, and those with cell position or pharynx unattached (Pun) phenotypes as “mild”. We used chi-square analysis to compare the frequency of each class for a given strain or condition. For RNA interference experiments with *pmr-1*, young adult hermaphrodites were injected with a *pmr-1* dsRNA construct mv ZK256.1a at 0.9 mg/ml using standard methods [95]. Injected worms were allowed to recover and lay eggs. The embryos of injected progeny were examined for levels of embryonic lethality and terminal phenotypes. For RNAi with *unc-68*, we used an RNAi feeding protocol [95].

### Temperature shift experiments

Embryos were extracted from gravid *pmr-1(ru5)* adults grown at the indicated temperature from the early-to-mid-L4 stage, shifted to the second temperature at the indicated time point, and then maintained at that second temperature for the duration of embryogenesis. Each embryo was scored for hatching and terminal phenotypes. Experiments at the two temperatures were calibrated based on comparisons of the timing of key developmental events.

### Lineaging and cell migration analysis

For lineaging and cell migration analysis, *pmr-1(ru5)* embryos carrying nuclear-localized GFP were imaged at 25°C according to the protocol outlined in [50], [52], [53]. Four-dimensional representations of embryos were generated using the Acetree program [51]. *pmr-1(ru5)* lineages (n = 6) were compared to controls (n = 6 to 8 ) (kindly provided by M. Boeck, P. Weisdepp, and R. Waterston). Files for each embryo were synchronized to the same developmental time points based on cell division patterns in several lineages. Because of the different migration patterns for the lineages tested, several techniques were used for measuring cell movement. To analyze ingression, the timing of the ingressions of P4, D, MS, and C-lineage daughters in N2 was compared to *pmr-1(ru5)* embryos. C-lineage cell migration was calculated by measuring the distance of the migrating cell nucleus relative to the ventral-most cell in the embryo. The average slopes of distance over time were used for comparisons. Ventral cell migration was calculated by measuring the distance of the migrating cell nucleus relative to the left or right edge of the embryo and total migration distances over time were compared. The position of the anterior–most cells was measured using polar or Cartesian coordinates, the positions plotted over time, and positions compared in each dimension. In all cases, t-tests were used to determine if the differences between the controls and *pmr-1(ru5)* embryos were statistically significant.

### Gene expression analysis

For analysis of the expression patterns of GFP-fusion proteins, control and *pmr-1(ru5)* embryos were grown at 25°C and compared at specific developmental stages. In some experiments, the position and number of nuclei expressing markers were analyzed using t-tests. For analysis using *in situ* immunofluorescence, control and *pmr-1* strains were collected and fixed using a freeze cracking technique and stained with anti-SMA-1, anti-NID-1, anti-AJM-1, anti-SQV-8, or anti-PMR-1 antisera [34], [62], [63], [66], [96]. Nomarski and fluorescent images of embryos were collected using an Olympus spinning disc microscope using Slidebook, Nikon E600 microscopes using a coolsnap CCD camera controlled by Metaview software, and Olympus DeltaVision deconvolution microscope (Applied Precision, Issaquah, WA); projected images were created using 4D macros within NIH Image and Image J.

## Supporting Information

Figure S1 The genomic location of *pmr-1* rescuing constructs and mutations. A. Genomic location of *pmr-1* on L.G. I. B. Position of the *pmr-1* gene, including the position of rescuing cosmids WRM0626dG10 and WRM066cH12, as well as cosmids C47B2 and CC4. WRM0622dA01 (not shown) is to the right of the region shown on this map. The dashed lines indicate the position of *pmr-1*, with *pmr-1* intron and exon structure for each of three major isoforms [33]. The *tm1750* allele has a 253 bp deletion upstream of exon 1 for *pmr-1*a/b but does alter *pmr-1*c. The *tm1840* allele removes the first exon and flanking sequencing of *pmr-1a/b.* The *jc10* allele has a premature stop, in exon 4 (Q451stop) of *pmr-1a/b*; *ru5* has an amino acid change (G142D) in exon 2 of *pmr-1b* (sequences of *tm1750* and *tm1840* alleles available at [46]). C. Complementation rescue data showing the cosmids and fosmids used for transformation into *pmr-1(ru5)*. After selecting for lines that stably expressed the co-transformation marker Rol6, lines were tested for significant growth at 25°C, demonstrating rescue of the *pmr-1(ru5)* mutant phenotype.
(TIF)

Figure S2 A one-hour pulse to the permissive temperature can rescue *pmr-1* mutant embryonic lethal phenotypes during anterior, C-lineage and ventral cell migrations. In temperature pulse experiments, embryos, extracted from gravid adults grown at the first temperature from the mid-L4 stage, were moved to the second temperature at the indicated time. At the end of a one-hour pulse, the embryos were returned to the original temperature for the remainder of embryogenesis and scored “viable” if they thrived past the L1 stage. Lines below the graph correspond to time of cell migration assays for anterior, C-derived, and ventral lineages. All times were normalized to correspond to development at 25°C; n = 4 to 23 embryos at each time point.
(TIF)

Figure S3 *pmr-1* mutant embryos show normal expression of cell fate markers. Control (A, C, E, G, I, K) and *pmr-1(ru5)* (B, C, F, H, J, L) embryos expressing cell fate markers AJM-1 (A, B), *pha-4* (C, D), *vab-7* (E, F), *hlh-1* (G, H), *plx-2* (I, J), and *kal-1* (K, L). Note that the position and number of cells expressing markers in *pmr-1(ru5)* mutant embryos is similar to that observed in controls, indicating normal acquisition of cell fates during development. However, some cells show positioning defects in *pmr-1(ru5*) mutant embryos, as in the anterior-most cells expressing AJM-1 (B, arrow) or in the C-lineage-derived muscles cells expressing *hlh-1* (H, arrows). A–D are comma stage embryos. E–H are gastrulation stage embryos. I–L are enclosure stage embryos. Strains, reagents, and references are as indicated in Table 3. Anterior is to the left; A–D are lateral views, E–L are ventral views.
(TIF)

Figure S4 *pmr-1* mutant embryos have normal cell polarity in polarized epithelial cells. The localization of proteins with distinctive polarized expression patterns is similar in control and *pmr-1(ru5)* mutant embryos, indicating the mutation does not cause a visible loss of cell polarity. PAR-6 is localized to apical membrane in the pharynx (A, B, arrow), AJM-1 is localized to apical adhesion junctions in the gut (C, D arrow) and SMA-1 is apically localized in the hypodermis (E, F, arrow) in both control (A, C, E) and *pmr-1(ru5)* mutant embryos (B, D, F), although we do see some altered expression due to mis-positioned anterior blastomeres in *pmr-1(ru5)* mutant embryos (arrowheads in B, D, F). The localization of AJM-1 to apical adhesion complexes (G, H, arrow) and the basal localization of NID-1 (I, J, arrow) in the pharynx are similar in control (G, I K) and *pmr-1(ru5)* mutant embryos (H, J, L). VAB-9 (O, Q) and MEL-11 (P, R) show normal co-localization with AJM-1 (M, N, Q, R) in the lateral hypodermis in *pmr-1(jc10)* mutant embryos (M–R). Even in ventral (M, arrowhead) or lateral (N, arrow) hypodermal cells with positioning defects, AJM-1, VAB-9 and MEL-11 (M–R) are properly localized to the apical adhesion junction. Strains, reagents, and references are as indicated in Table 3; all strains grown at 25°C. Anterior is to the left in all figures; lateral views. Embryos are comma stage (A–F) or elongation stage (G–R).
(TIF)
