# Supplementary figures and images for: The Secretory Pathway Calcium ATPase PMR-1/SPCA1 Has Essential Roles in Cell Migration during Caenorhabditis elegans Embryonic Development
Source: PLoS Genet. 2013 May 16;9(5):e1003506. doi: 10.1371/journal.pgen.1003506 (PMC3656159; doi:10.1371/journal.pgen.1003506)

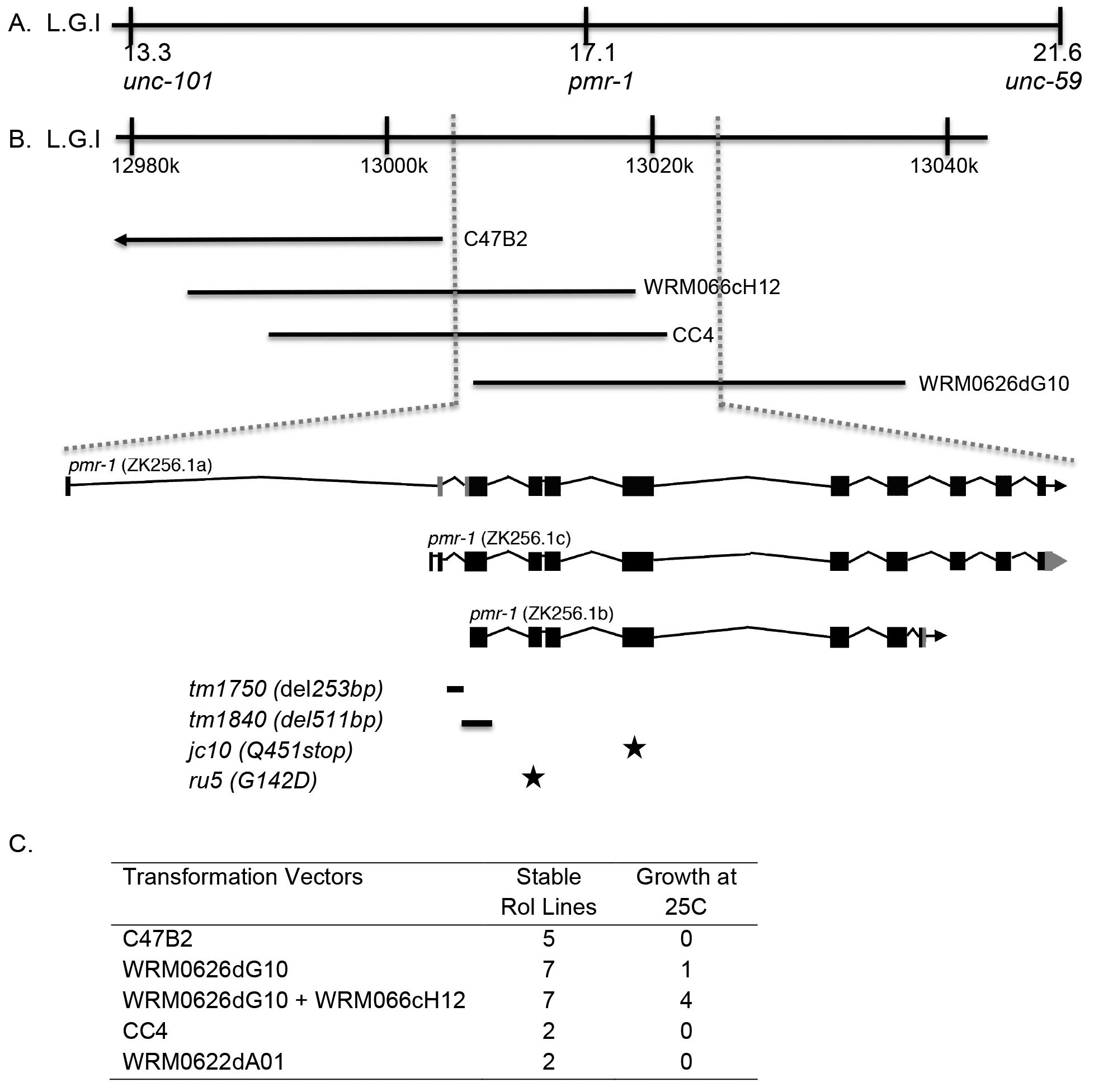

Supplement: Figure S1 — The genomic location of pmr-1 rescuing constructs and mutations. A. Genomic location of pmr-1 on L.G. I. B. Position of the pmr-1 gene, including the position of rescuing cosmids WRM0626dG10 and WRM066cH12, as well as cosmids C47B2 and CC4. WRM0622dA01 (not shown) is to the right of the region shown on this map. The dashed lines indicate the position of pmr-1, with pmr-1 intron and exon structure for each of three major isoforms [33]. The tm1750 allele has a 253 bp deletion upstream of exon 1 for pmr-1a/b but does alter pmr-1c. The tm1840 allele removes the first exon and flanking sequencing of pmr-1a/b. The jc10 allele has a premature stop, in exon 4 (Q451stop) of pmr-1a/b; ru5 has an amino acid change (G142D) in exon 2 of pmr-1b (sequences of tm1750 and tm1840 alleles available at [46]). C. Complementation rescue data showing the cosmids and fosmids used for transformation into pmr-1(ru5). After selecting for lines that stably expressed the co-transformation marker Rol6, lines were tested for significant growth at 25°C, demonstrating rescue of the pmr-1(ru5) mutant phenotype. (TIF) [file pgen.1003506.s001.tif]

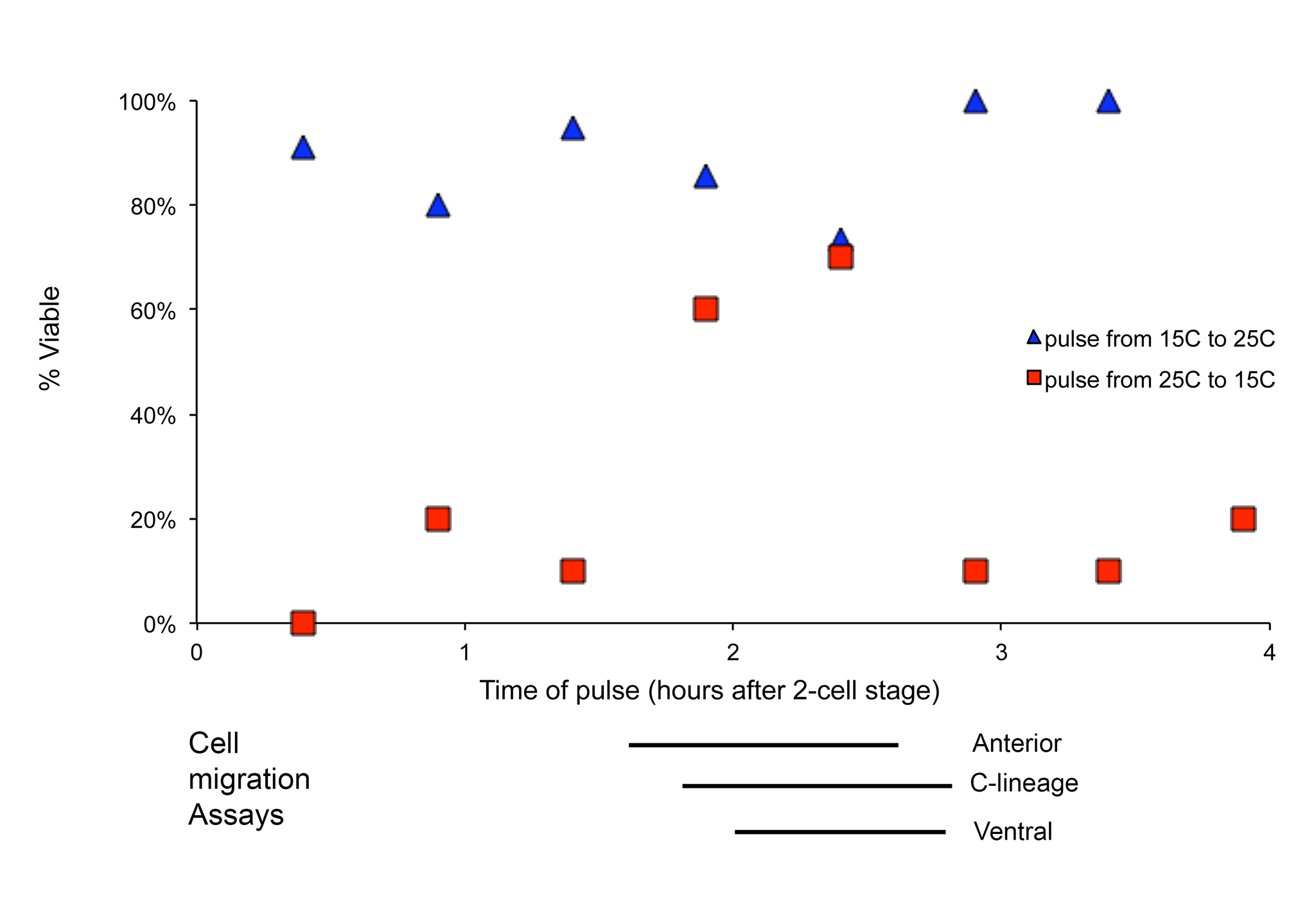

Supplement: Figure S2 — A one-hour pulse to the permissive temperature can rescue pmr-1 mutant embryonic lethal phenotypes during anterior, C-lineage and ventral cell migrations. In temperature pulse experiments, embryos, extracted from gravid adults grown at the first temperature from the mid-L4 stage, were moved to the second temperature at the indicated time. At the end of a one-hour pulse, the embryos were returned to the original temperature for the remainder of embryogenesis and scored “viable” if they thrived past the L1 stage. Lines below the graph correspond to time of cell migration assays for anterior, C-derived, and ventral lineages. All times were normalized to correspond to development at 25°C; n = 4 to 23 embryos at each time point. (TIF) [file pgen.1003506.s002.tif]

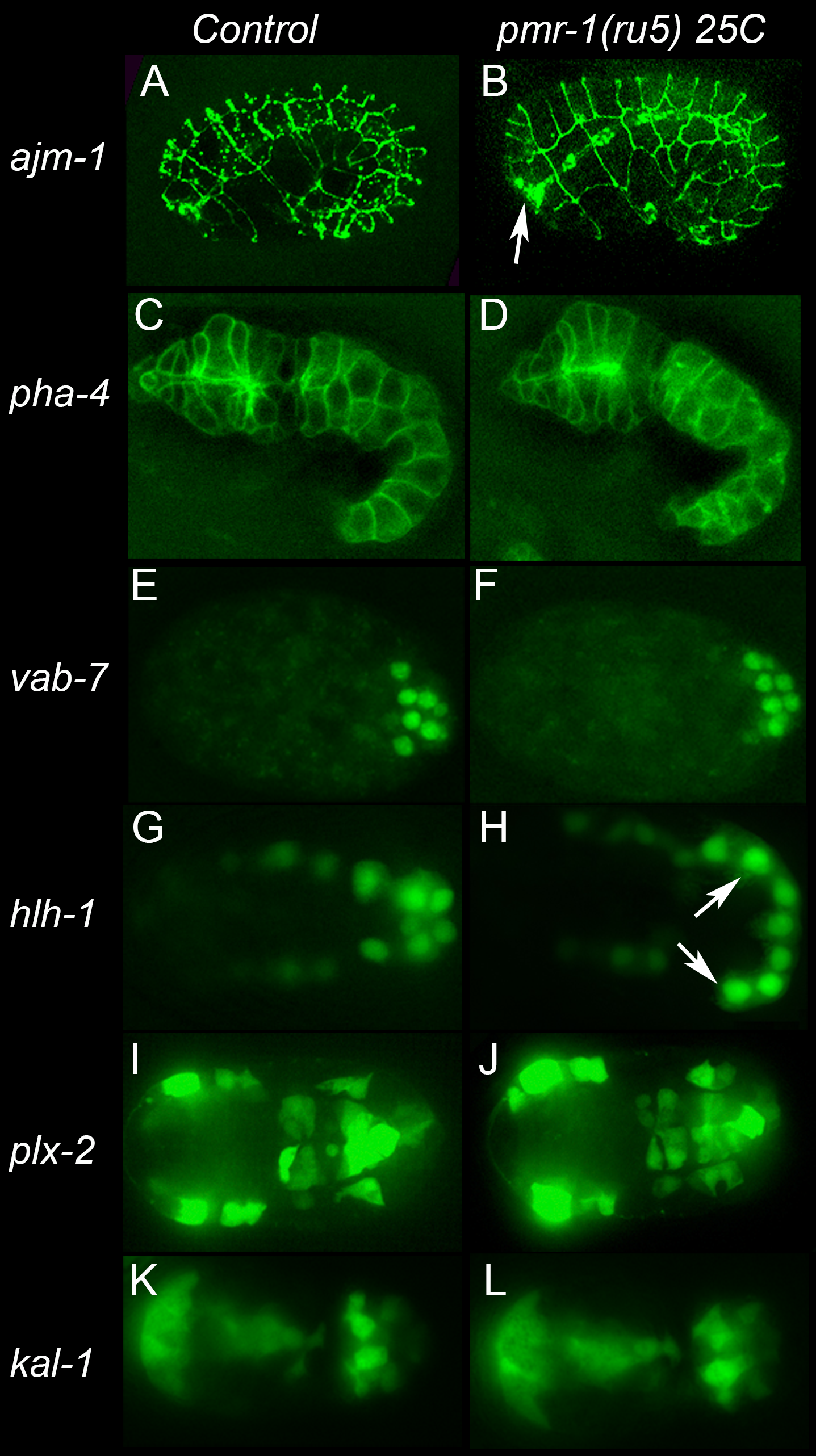

Supplement: Figure S3 — pmr-1 mutant embryos show normal expression of cell fate markers. Control (A, C, E, G, I, K) and pmr-1(ru5) (B, C, F, H, J, L) embryos expressing cell fate markers AJM-1 (A, B), pha-4 (C, D), vab-7 (E, F), hlh-1 (G, H), plx-2 (I, J), and kal-1 (K, L). Note that the position and number of cells expressing markers in pmr-1(ru5) mutant embryos is similar to that observed in controls, indicating normal acquisition of cell fates during development. However, some cells show positioning defects in pmr-1(ru5) mutant embryos, as in the anterior-most cells expressing AJM-1 (B, arrow) or in the C-lineage-derived muscles cells expressing hlh-1 (H, arrows). A–D are comma stage embryos. E–H are gastrulation stage embryos. I–L are enclosure stage embryos. Strains, reagents, and references are as indicated in Table 3. Anterior is to the left; A–D are lateral views, E–L are ventral views. (TIF) [file pgen.1003506.s003.tif]

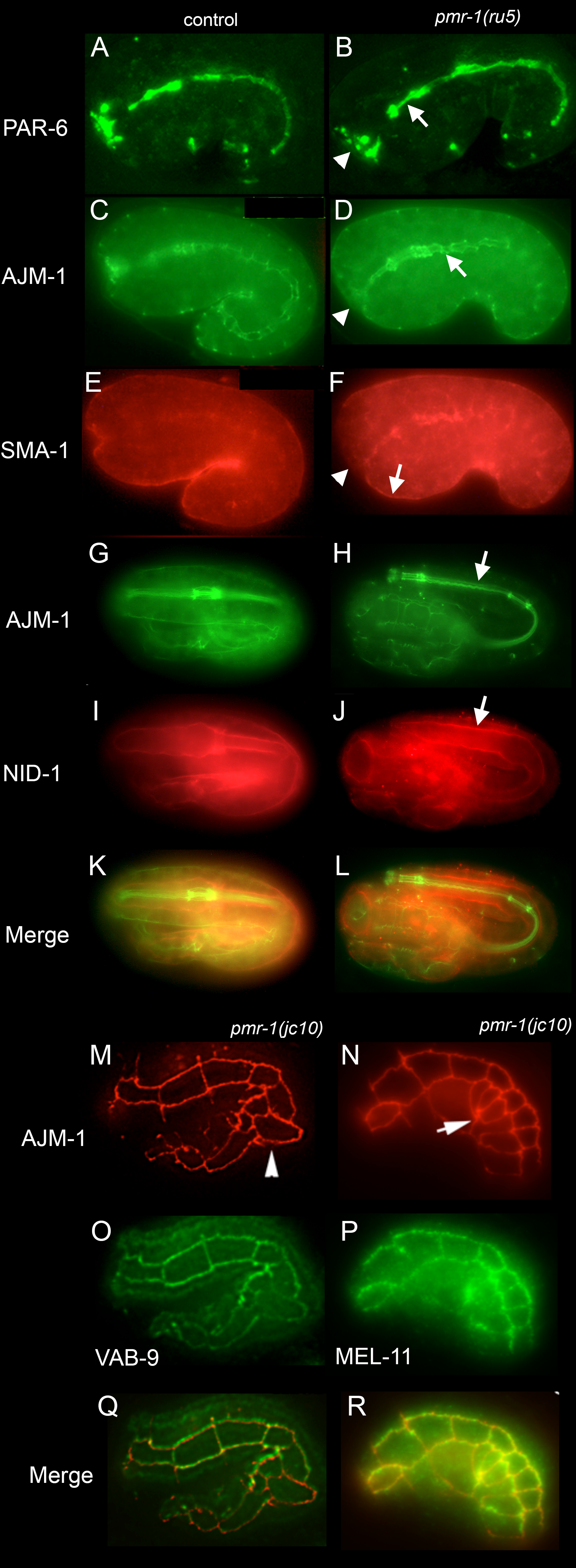

Supplement: Figure S4 — pmr-1 mutant embryos have normal cell polarity in polarized epithelial cells. The localization of proteins with distinctive polarized expression patterns is similar in control and pmr-1(ru5) mutant embryos, indicating the mutation does not cause a visible loss of cell polarity. PAR-6 is localized to apical membrane in the pharynx (A, B, arrow), AJM-1 is localized to apical adhesion junctions in the gut (C, D arrow) and SMA-1 is apically localized in the hypodermis (E, F, arrow) in both control (A, C, E) and pmr-1(ru5) mutant embryos (B, D, F), although we do see some altered expression due to mis-positioned anterior blastomeres in pmr-1(ru5) mutant embryos (arrowheads in B, D, F). The localization of AJM-1 to apical adhesion complexes (G, H, arrow) and the basal localization of NID-1 (I, J, arrow) in the pharynx are similar in control (G, I K) and pmr-1(ru5) mutant embryos (H, J, L). VAB-9 (O, Q) and MEL-11 (P, R) show normal co-localization with AJM-1 (M, N, Q, R) in the lateral hypodermis in pmr-1(jc10) mutant embryos (M–R). Even in ventral (M, arrowhead) or lateral (N, arrow) hypodermal cells with positioning defects, AJM-1, VAB-9 and MEL-11 (M–R) are properly localized to the apical adhesion junction. Strains, reagents, and references are as indicated in Table 3; all strains grown at 25°C. Anterior is to the left in all figures; lateral views. Embryos are comma stage (A–F) or elongation stage (G–R). (TIF) [file pgen.1003506.s004.tif]
